# Analysing Standard Progressive Matrices (SPM-LS) with Bayesian Item Response Models

**DOI:** 10.3390/jintelligence8010005

**Published:** 2020-02-04

**Authors:** Paul-Christian Bürkner

**Affiliations:** Department of Computer Science, Aalto University, Konemiehentie 2, 02150 Espoo, Finland; paul.buerkner@gmail.com

**Keywords:** Standard Progressive Matrices, Item Response Theory, Bayesian statistics, brms, Stan, R

## Abstract

Raven’s Standard Progressive Matrices (SPM) test and related matrix-based tests are widely applied measures of cognitive ability. Using Bayesian Item Response Theory (IRT) models, I reanalyzed data of an SPM short form proposed by Myszkowski and Storme (2018) and, at the same time, illustrate the application of these models. Results indicate that a three-parameter logistic (3PL) model is sufficient to describe participants dichotomous responses (correct vs. incorrect) while persons’ ability parameters are quite robust across IRT models of varying complexity. These conclusions are in line with the original results of Myszkowski and Storme (2018). Using Bayesian as opposed to frequentist IRT models offered advantages in the estimation of more complex (i.e., 3–4PL) IRT models and provided more sensible and robust uncertainty estimates.

## 1. Introduction

Raven’s Standard Progressive Matrices (SPM) test (Raven 1941) matrix-based tests are widely applied measures of cognitive ability (e.g., Jensen et al. 1988; Pind et al. 2003). Due to their non-verbal content, which reduces biases due to language and cultural differences, they are considered one of the purest measures of fluid intelligence (Myszkowski and Storme 2018). However, a disadvantage of the original SPM is that its administration takes considerable time as 60 items have to be answered and time limits are either very loose or not imposed at all (e.g., Pind et al. 2003). Thus, using it as part of a bigger procedure involving the administration of the SPM as part of a battery of tests and/or experiments may be problematic. This is not only due to direct time restrictions but also because participants’ motivation and concentration tends to decline over the course of the complete procedure, potentially leading to less valid measurements (e.g., Ackerman and Kanfer 2009).

Recently, Myszkowski and Storme (Myszkowski and Storme 2018) proposed a short version of the original SPM test, called SPM-LS, comprising only the last block of the 12 most complex SPM items. They evaluated the statistical properties of the SPM-LS using methods of Item Response Theory (IRT). IRT is widely applied in the human sciences to model persons’ responses on a set of items measuring one or more latent constructs (for a comprehensive introduction see Embretson and Reise 2013; Lord 2012; van der Linden and Hambleton 1997). Due to its flexibility compared to Classical Test Theory (CTT), IRT provides the formal statistical basis for most modern psychological measurement. The best known IRT models are likely those for binary responses, which predict the probability of a correct answer depending on item’s properties and the participant’s latent abilities. As responses on SPM items can be categorized as either right or wrong, I focus on these binary models in the present paper (although other models for these data are possible as well; see Myszkowski and Storme 2018). Myszkowski and Storme (Myszkowski and Storme 2018), whose data I sought to reanalyze, used frequenstist IRT models for inference. In this paper, I apply Bayesian IRT models instead and investigate potential differences to the original results. In doing so, I hope to improve our understanding of the robustness of the inference obtainable from the SPM-LS test and to illustrate the application of Bayesian IRT methods.

## 2. Bayesian IRT Models

In Bayesian statistics applied to IRT, we aim to estimate the posterior distribution p(θ,ξ|y) of the person and item parameters (θ and ξ, respectively, which may vary in number depending on the model) given the data *y*. We may be either interested in the posterior distribution directly, or in quantities that can be computed on its basis. The posterior distribution for an IRT model is defined as

(1) $$p\left(\theta ,\xi |y\right)=\frac{p(y|\theta ,\xi )\;p(\theta ,\xi )}{p(y)}.$$

In the above equation, p(y|θ,ξ) is the likelihood, p(θ,ξ) is the prior distribution, and p(y) is the marginal likelihood. The likelihood p(y|θ,ξ) is the distribution of the data given the parameters and thus relates the data to the parameters. tThe prior distribution p(θ,ξ) describes the uncertainty in the person and item parameters before having seen the data. It thus allows explicitly incorporating prior knowledge into the model and/or helping to identify the model. In practice, we factorize the joint prior p(θ,ξ) into the product of p(θ) and p(ξ) so that we can specify priors on person and items parameters independently. I provide more details on likelihoods and priors for Bayesian IRT models in the next section. The marginal likelihood p(y) serves as a normalizing constant so that the posterior is an actual probability distribution. Except in the context of specific methods (e.g., Bayes factors), p(y) is rarely of direct interest.

Obtaining the posterior distribution analytically is only possible in certain cases of carefully chosen combinations of prior and likelihood, which may considerably limit modelling flexibility but yield a computational advantage. However, with the increased power of today’s computers, Markov-Chain Monte-Carlo (MCMC) sampling methods constitute a powerful and feasible alternative to obtaining posterior distributions for complex models in which the majority of modeling decisions is made based on theoretical and not computational grounds. Despite all the computing power, these sampling algorithms are computationally very intensive and thus fitting models using full Bayesian inference is usually much slower than in point estimation techniques. If using MCMC to fit a Bayesian model turns out to be infeasible, an alternative is to perform optimization over the posterior distribution to obtain Maximum A-Posteriori (MAP) estimates, a procedure similar to maximum likelihood estimation just with additional regularization through priors. MCMC and MAP estimates differ in at least two aspects. First, MCMC allows obtaining point estimates (e.g., means or medians) from the unidimensional marginal posteriors of the quantities of interest, which tend to be more stable than MAP estimates obtained from the multidimensional posterior over all parameters. Second, in contrast to MAP, MCMC provides a set of random draws from the model parameters’ posterior distribution. After the model fitting, the posterior distribution of any quantity that is a function of the original parameters can be obtained by applying the function on a draw by draw basis. As such, the uncertainty in the posterior distribution naturally propagates to new quantities, a highly desirable property that is difficult to achieve using point estimates alone.

In the present paper, I apply Bayesian binary IRT models to the SPM-LS data using both MCMC and MAP estimators. Their results are compared to those obtained by frequentist maximum likelihood estimation. For a comprehensive introduction to Bayesian IRT modeling see, for example, the works of Fox (Fox 2010), Levy and Mislevy (Levy and Mislevy 2017), and Rupp, Dey, and Zumbo (Rupp et al. 2004).

### 2.1. Bayesian IRT Models for Binary Data

In this section, I introduce a set of Bayesian IRT models for binary data and unidimensional person traits. Suppose that, for each person *j* (j=1,…,J) and item *i* (i=1,…,I), we have observed a binary response $y_{ji}$, which is coded as 1 for a correct answer and 0 otherwise. With binary IRT models, we aim to model $p_{ji}=P\left(y_{ji}=1\right)$, that is, the probability the person *j* answers item *i* correctly. In other words, we assume a Bernoulli distribution for the responses $y_{ji}$ with success probability $p_{ji}$:

(2) $$y_{ji}∼\mathrm{Bernoulli}\left(p_{ji}\right)$$

Across all models considered here, we assume that all items measure a single latent person trait $\theta_{j}$. For the present data, we can expect $\theta_{j}$ to represent something closely related to fluid intelligence (Myszkowski and Storme 2018). The most complex model I consider in this paper is the four-parameter logistic (4PL) model and all other simpler models result from this model by fixing some item parameters to certain values. In recent years, the 4PL model has received much attention in IRT research due to its flexibility in modeling complex binary response processes (e.g., Culpepper 2016, 2017; Loken and Rulison 2010; Waller and Feuerstahler 2017). Under this model, we express $P(y_{ji}=1)$ via the equation

(3) $$P\left(y_{ji}=1\right)=\gamma_{i}+\left(1-\gamma_{i}-\psi_{i}\right)\frac{1}{1+\exp(-\left(\beta_{i}+\alpha_{i}\theta_{j}\right))}.$$

In the 4PL model, each item has four associated item parameters. The $\beta_{i}$ parameter describes the location of the item, that is, how easy or difficult it is in general. In the above formulation of the model, higher values of $\beta_{i}$ imply higher success probabilities and hence $\beta_{i}$ can also be called the “easiness” parameter. The $\alpha_{i}$ parameter describes how strongly item *i* is related to the latent person trait $\theta_{j}$. We can call $\alpha_{i}$ “factor loading”, “slope”, or “discrimination” parameter, but care must be taken that none of these terms is used uniquely and their exact meaning can only be inferred in the context of a specific model (e.g., see Bürkner 2019 for a somewhat different use of the term “discrimination” in IRT models). For our purposes, we assume $\alpha_{i}$ to be positive as we expect answering the items correctly implies higher trait scores than when answering incorrectly. In addition, if we did not fix the sign of $\alpha_{i}$, we may run into identification issues as changing the sign of $\alpha_{i}$ could be compensated by changing the sign of $\theta_{j}$ without a change in the likelihood.

The $\gamma_{i}$ parameter describes the guessing probability, that is, the probability of any person answering item *i* correctly even if they do not know the right answer and thus have to guess. For obvious reasons, guessing is only relevant if the answer space is reasonably small. In the present data, participants saw a set of 8 possible answers of which exactly one was considered correct. Thus, guessing cannot be ruled out and would be equal to $\gamma_{i}=1/8$ for each item if all answer alternatives had a uniform probability to be chosen given that a person guesses. Lastly, the $\psi_{i}$ parameter enables us to model the possibility that a participant makes a mistake even though they know the right answer, perhaps because of inattention or simply misclicking when selecting the chosen answer. We may call $\psi_{i}$ the “lapse”, “inattention”, or “slipping” parameter. Usually, these terms can be used interchangeably but, as always, the exact meaning can only be inferred in the context of the specific model. As the answer format in the present data (i.e., “click on the right answer”) is rather simple and participants have unlimited time for each item, mistakes due to lapses are unlikely to appear. However, by including a lapse parameter into our model, we are able to explicitly check whether lapses played a substantial role in the answers.

We can now simplify the 4PL model in several steps to yield the other less complex models. The 3PL model results from the 4PL model by additionally fixing the lapse probability to zero, that is, $\psi_{i}=0$ for all items. In the next step, we can obtain the 2PL model from the 3PL model by also fixing the guessing probabilities to zero, that is, $\gamma_{i}=0$ for all items. In the last simplification step, we obtain the 1PL model (also known as Rasch model Rasch 1961) from the 2PL model by assuming the factor loadings to be one, that is, $\alpha_{i}=1$ for all items. Even though didactically I find it most intuitive and helpful to introduce the models from most to least complex, I recommend the inverse order in applications, that is, starting from the simplest (but still sensible) model. The reason is that more complex models tend to be more complicated to fit in the sense that they both take longer (especially when using MCMC estimation) and yield more convergence problems (e.g., Bürkner 2019; Gelman et al. 2013). If we started by fitting the most complex model and, after considerable waiting time, found the model to not have converged, we may have no idea which of the several model components were causing the problem(s). In contrast, by starting simple and gradually building towards more complex models, we can make sure that each model component is reasonably specified and can be reliably estimated before we move further. As a result, when a problem occurs, we are likely to have much clearer understanding of why/where it occurred and how to fix it.

With the model likelihood fully specified by Equations (2) and (3) (potentially with some fixed item parameters), we are, in theory, already able to obtain estimates of person and item parameters via maximum likelihood (ML) estimation. However, there are multiple potential issues that can get into our way at this point. First, we simply may not have enough data to obtain sensible parameter estimates. As a rule of thumb, the more complex a model, the more data we need to obtain the same estimation precision. Second, there may be components in the model which will not be identified no matter how much data we add. An example would be binary IRT models from 2PL upwards as (without additional structure) we cannot identify the scale of both $\theta_{j}$ and $\alpha_{i}$ at the same time. This is because, due to the multiplicative relationship, multiplying one of the two by a constant can be adjusted for by dividing the other by the same constant without changing the likelihood. Third, we need to have software that is able to do the model fitting for us, unless we want to hand code every estimation algorithm on our own. Using existing software requires (re)expressing our models in a way the software understands. I will focus on the last issue first and then address the former two.

### 2.2. IRT Models as Regression Models

There are a lot of IRT specific software packages available, in particular in the programming language R (R Core Team 2019), for example, mirt (Chalmers 2012), sirt (Robitzsch 2019), or TAM (Robitzsch et al. 2019; see Bürkner 2019 for a detailed comparison). In addition to these more specialized packages, general purpose probabilistic programming languages can be used to specify and fit Bayesian IRT models, for example, BUGS (Lunn et al. 2009; see also Curtis 2010), JAGS (Plummer 2013; see also Depaoli et al. 2016; Zhan et al. 2019), or Stan (Carpenter et al. 2017; see also Allison and Au 2018; Luo and Jiao 2018). In this paper, I use the brms package (Bürkner 2017, 2018), a higher level interface to Stan, which is not focused specifically on IRT models but more generally on (Bayesian) regression models. Accordingly, we need to rewrite our IRT models in a form that is understandable for brms or other packages focussed on regression models.

The first implication of this change of frameworks is that we now think of the data in long format, with all responses from all participants on all items in the same data column coupled with additional columns for person and item indicators. That is, $y_{ji}$ is now formally written as $y_{n}$ where *n* is the observation number ranging from 1 to N=J×I. If we needed to be more explicit we could also use $y_{j_{n}i_{n}}$ to indicate that each observation number *n* has specific indicators *j* and *i* associated with it. The same goes for item and person parameters. For example, we may write $\theta_{n_{j}}$ to refer to the ability parameter of the person *j* to whom the *n*th observation belongs.

One key aspect of regression models is that we try to express parameters on an unconstrained space that spans the whole real line. This allows for using linear (or more generally additive) predictor terms without having to worry about whether these predictor terms fulfill certain boundaries, for instance, are positive or within the unit interval [0,1]. In the considered binary IRT models, we need to ensure that the factor loadings α are positive and that guessing and lapse parameters, γ and ψ, respectively, are within [0,1] as otherwise the interpretation of the latter two as probabilities would not be sensible. To enforce these parameter boundaries within a regression, we apply (inverse-)link functions. That is, for α, we use the log-link function (or equivalently the exponential response function) so that

(4) $$\alpha =\exp(\eta_{\alpha })$$

where $\eta_{\alpha_{n}}$ is unconstrained. Similarly, for γ and ψ, we use the logit-link (or equivalently the logistic response function) so that  

(5) $$\begin{array}{cc} \gamma & =\mathrm{logistic}\left(\eta_{\gamma }\right)=\frac{1}{1+\exp(-\eta_{\gamma })}, \end{array}$$

(6) $$\begin{array}{cc} \psi & =\mathrm{logistic}\left(\eta_{\psi }\right)=\frac{1}{1+\exp(-\eta_{\psi })} \end{array}$$

where $\eta_{\gamma }$ and $\eta_{\psi }$ are unconstrained. The location parameters β are already unbounded and as such do not need an additional link function so that simply $\beta =\eta_{\beta }$. The same goes for the ability parameters θ. On the scale of the linear predictors, we can perform the usual regression operations, perhaps most importantly modeling predictor variables or including multilevel structure. In the present data, we do not have any additional person or item variables available so there are no such predictors in our models (but see Bürkner 2019 for examples if you are interested in this option). However, there certainly is multilevel structure as we have both multiple observations per item and per person, which we seek to model appropriately, as detailed in the next section.

### 2.3. Model Priors and Identification

When it comes to the specification of priors on item parameters, we typically distinguish between non-hierarchical and hierarchical priors (Bürkner 2019; Fox 2010; Levy and Mislevy 2017) with the former being applied more commonly (e.g., Bürkner 2018; Levy and Mislevy 2017). When applying non-hierarchical priors, we directly equate the linear predictor η (for any of the item parameter classes) with item-specific parameters $b_{i}$, so that

(7) $$\eta_{n}=b_{i_{n}}$$

for each observation *n* and corresponding item *i*. Since η is on an unconstrained scale so are the $b_{i}$ parameters and we can apply location-scale priors such as the normal distribution with mean μ and standard deviation σ:

(8) $$b_{i}∼\mathrm{normal}\left(\mu ,\sigma \right)$$

In non-hierarchical priors, we fix μ and σ to sensible values. In general, priors can only be understood in the context of the model as a whole, which renders general recommendation for prior specification difficult (Gelman et al. 2017). If we only use our understanding of the scale of the modeled parameters without any data-specific knowledge, we arrive at weakly-informative prior distributions. By weakly-informative I mean penalizing a-priori implausible values (e.g., a location parameter of 1000 on the logit-scale) without affecting the a-priori plausible parameter space too much (e.g., location parameters within the interval [-3,3] on the logit-scale). Weakly informative normal priors are often centered around μ=0 with σ appropriately chosen so that the prior covers the range of plausible parameter values but flattens out quickly outside of that space. For more details on priors for Bayesian IRT models, see the works of Bürkner (Bürkner 2019), Fox (Fox 2010), and Levy and Mislevy (Levy and Mislevy 2017).

A second class of priors for item parameters are hierarchical priors. For this purpose, we apply the non-centered parameterization of hierarchical models (Gelman et al. 2013) as detailed in the following. We split the linear predictor η (for any of the item parameter classes) into an overall parameter, $\bar{b}$, and an item-specific deviation from the overall parameter, ${\tilde{b}}_{i}$, so that

(9) $$\eta_{n}=\bar{b}+{\tilde{b}}_{i_{n}}$$

Without additional constraints, this split is not identified as adding a constant to the overall parameter can be compensated by subtracting the same constant from all ${\tilde{b}}_{i}$ without changing the likelihood. In Bayesian multilevel models, we approach this problem by specifying a hierarchical prior on ${\tilde{b}}_{i}$ via

(10) $${\tilde{b}}_{i}∼\mathrm{normal}\left(0,\sigma \right)$$

where σ is the standard deviation parameter over items on the unconstrained scale. Importantly, not only ${\tilde{b}}_{i}$ but also the hyperparameters $\bar{b}$ and σ are estimated during the model fitting.

Using the prior distribution from (10), we would assume the item parameters of the same item to be unrelated but, in practice, it is quite plausible that they are intercorrelated (Bürkner 2019). To account for such (linear) dependency, we can extend Equation (10) to the multivariate case, so that we can model the vector ${\tilde{b}}_{i}=\left({\tilde{b}}_{\beta_{i}},{\tilde{b}}_{\alpha_{i}},{\tilde{b}}_{\gamma_{i}},{\tilde{b}}_{\psi_{i}}\right)$ jointly via a multivariate normal distribution:

(11) $${\tilde{b}}_{i}∼\mathrm{multinormal}\left(0,\sigma ,\Omega \right)$$

where $\sigma =(\sigma_{\beta },\sigma_{\alpha },\sigma_{\gamma },\sigma_{\psi })$ is the vector of standard deviations and Ω is the correlation matrix of the item parameters (see also Bürkner 2017, 2019; Nalborczyk 2019). To complete the prior specification for the item parameters, we need to set priors on $\bar{b}$ and σ. For this purpose, weakly-informative normal prior on $\bar{b}$ and half-normal priors on σ are usually fine but other options are possible as well (see Bürkner 2019 for details).

A decision between hierarchical and non-hierarchical priors is not always easy. If in doubt, one can try out both kinds of priors and investigate whether they make a relevant difference. Personally, I prefer hierarchical priors as they imply some data-driven shrinkage due to their scale being learned by the model on the fly. In addition, they naturally allow item parameters to share information across parameter classes via the correlation matrix Ω.

With respect to the person parameters, it is most common to apply hierarchical priors of the form

(12) $$\theta_{j}∼\mathrm{normal}\left(0,\sigma_{\theta }\right)$$

where, similar as for hierarchical priors on item parameters, $\sigma_{\theta }$ is a standard deviation parameter estimated as part of the model on which we put a weakly-informative prior. To give the reader intuition: With the overall effects in our model, we model the probability that an average person (with an ability of zero, thus imagine the ability to be centered) answers an average item (with all item parameters at their average values which we estimate). The varying effects then give us the deviations from the average person or item, so that we can “customize” our prediction of the solution probability to more or less able persons, more or less easy items, more or less discriminatory items, etc.

In 2PL or more complex models, we can also fix $\sigma_{\theta }$ to some value (usually 1) as the scale is completely accounted for by the scale of the factor loadings $\sigma_{\alpha }$. However, when using weakly-informative priors on both θ and α as well as on their hyperparameters, estimating $\sigma_{\theta }$ actually poses no problem for model estimation. Importantly, however, we do not include an overall person parameter $\bar{\theta }$ as done for item parameters in (9) as this would conflict with the overall location parameter ${\bar{b}}_{\beta }$ leading to substantial convergence problems in the absence very informative priors. This does not limit the model’s usefulness as only differences of person parameters are of relevance, not their absolute values on an (in principal) arbitrary latent scale.

## 3. Analysis of the SPM-LS Data

The Bayesian IRT models presented above were applied to the SPM data of Myszkowski and Storme (Myszkowski and Storme 2018). The analyzed data consist of responses from 499 participants on the 12 most difficult SPM items and are freely available online (https://data.mendeley.com/datasets/h3yhs5gy3w/1). The data gathering procedure was described in detail by Myszkowski and Storme (Myszkowski and Storme 2018). Analyses were performed in R (R Core Team 2019) using brms (Bürkner 2017) and Stan (Carpenter et al. 2017) for model specification and estimation via MCMC. To investigate potential differences between hierarchical and non-hierarchical priors on the item parameters, models were estimated for both of these priors. Below, I refer to these approaches as hierarchical MCMC (MCMC-H) and non-hierarchical MCMC (MCMC-NH). Priors on person parameters were always hierarchical and weakly informative priors were imposed on the remaining parameters. All considered models converged well according to sample-agnostic (Vehtari 2019) and sampler-specific (Betancourt 2017) diagnostics. In the presentation of the results below, I omit details of prior distributions and auxiliary model fitting arguments. All details and the fully reproducible analysis are available on GitHub (https://github.com/paul-buerkner/SPM-IRT-models).

In addition to estimating the IRT models using MCMC, I also fitted the models via optimization as implemented in the mirt package (Chalmers 2012). Here, I considered two options: (1) a fully frequentist approach maximizing the likelihood under the same settings as in the original analysis of Myszkowski and Storme (Myszkowski and Storme 2018); and (2) a Bayesian optimization approach where I imposed the same priors on item parameters as in MCMC-NH. I refer to these two methods as maximum likelihood (ML) and maximum a-posteriori (MAP), respectively. For models involving latent variables, such as IRT models, ML or MAP optimization have to be combined with numerical integration over the latent variables as the mode of the joint distribution of all parameters including latent variables does not exist in general (e.g., see Bates et al. 2015). Such a combination of optimization and integration is commonly referred to as expectation-maximization (EM). A thorough discussion on EM methods is outside the scope of the present paper but the interested reader is referred to the work of Do and Batzoglou (Do and Batzoglou 2008).

### 3.1. Model Estimation

For estimation in a multilevel regression framework such as the one of brms, the data need to be represented in long format. In the SPM-LS data, the relevant variables are the binary response of the participants (variable response2) coded as either correct (1) or incorrect (0) as well as person and item identifiers. Following the principal of building models bottom-up, I start with the estimation of the most simple sensible model, that is, the 1PL model. When both person and item parameters are modeled hierarchically, the brms formula for the 1PL model can be specified as 
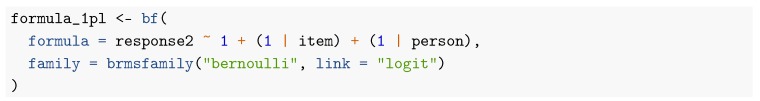


**Figure 1:**
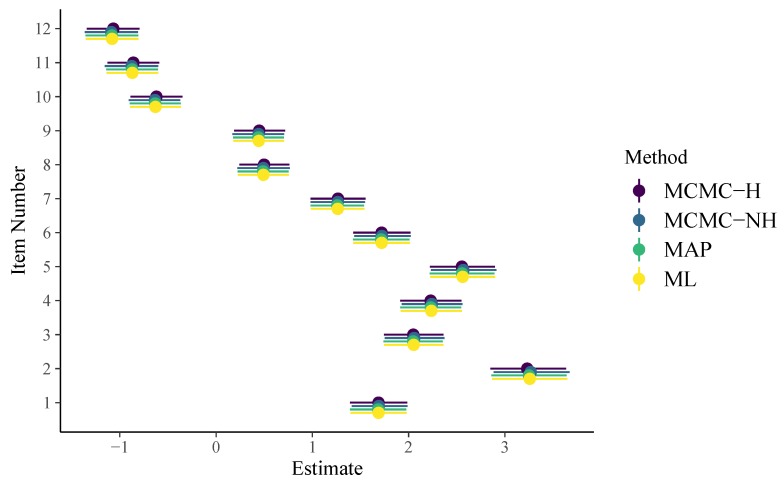
Item parameters of the 1PL model. Horizontal lines indicate 95% uncertainty intervals.

**Figure 2:**
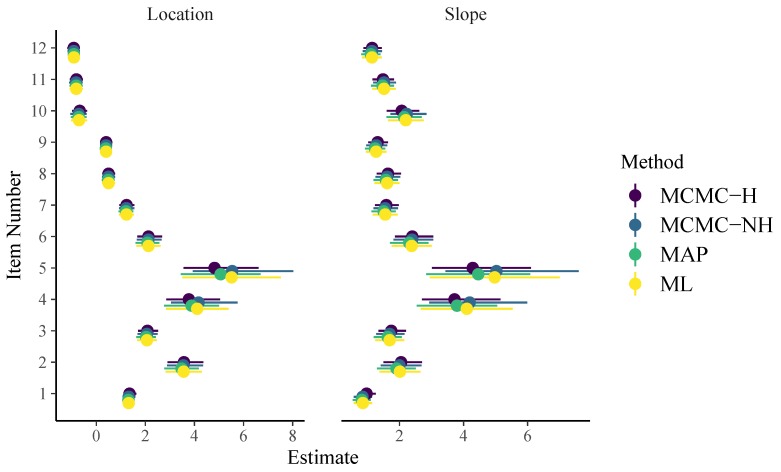
Item parameters of the 2PL model. Horizontal lines indicate 95% uncertainty intervals.

**Figure 3:**
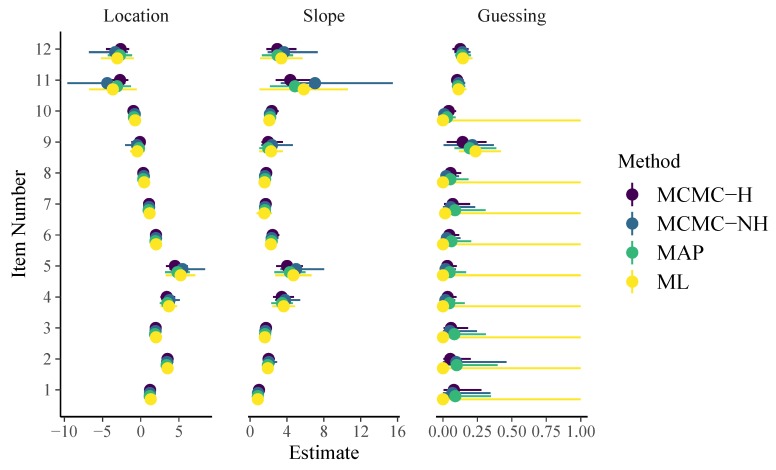
Item parameters of the 3PL model. Horizontal lines indicate 95% uncertainty intervals.

To apply non-hierarchical item parameters, we have to use the formula response2 ~ 0 + item + (1 | person) instead (see the code on Github for more details). For a thorough introduction and discussion of the brms formula syntax, see Bürkner (2017, 2018, 2019). As displayed in Figure 1, item parameter estimates of all methods are very similar for the 1PL model. In addition, their uncertainty estimates align closely as well. The brms formula for the 2PL model looks as follows: 
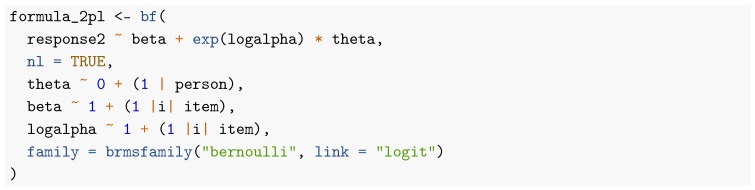


When comparing the formulas for the 1PL and 2PL models, we see that the structure has changed considerably as a result of going from a generalized linear model to a generalized non-linear model (see Bürkner 2019 for more details). As displayed in Figure 2, item parameter point and uncertainty estimates of all methods are rather similar for the 2PL model but not as close as for the 1PL model. In particular, we see that the slope estimates of Items 4 and 5 vary slightly, presumably due to different amounts of regularization implied by the priors. The brms formula for the 3PL model looks as follows: 
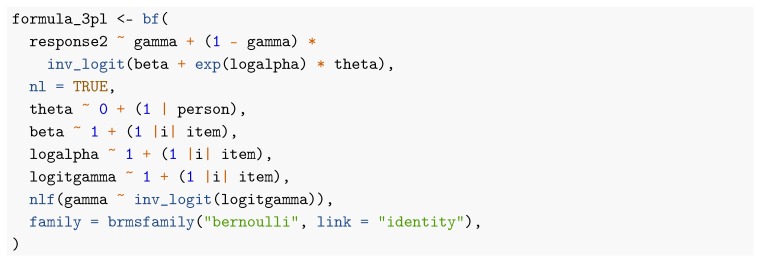


Note that, in the family argument, we now use link = "identity" instead of link = "logit" and build the logit link directly into the formula via inv_logit(beta + exp(logalpha) * theta). This is necessary to correctly include guessing parameters (Bürkner 2019). As displayed in Figure 3, item parameter estimates of all methods are still quite similar when it comes to locations and slopes of the 3PL model. However, guessing parameter estimates are quite different: ML obtains point estimates of 0 for all but three items with uncertainty intervals ranging the whole definition space from 0 to 1. This is caused by an artifact in the computation of the approximate standard errors because point estimates are located at the boundary of the parameter space at which maximum likelihood theory does not hold. In contrast, point estimates of guessing parameters as obtained by all regularized models are close to but not exactly zero for most items and corresponding uncertainty estimates appear more realistic (i.e., much narrower) than those obtained by pure ML.

On Github, I also report results for the 3PL model with guessing probabilities fixed to 1/8 derived under the assumptions that, in the case of guessing, all alternatives are equally likely. According to Figure 3 and model comparisons shown on GitHub, this assumption does not seem to hold for the present data.

**Figure 4:**
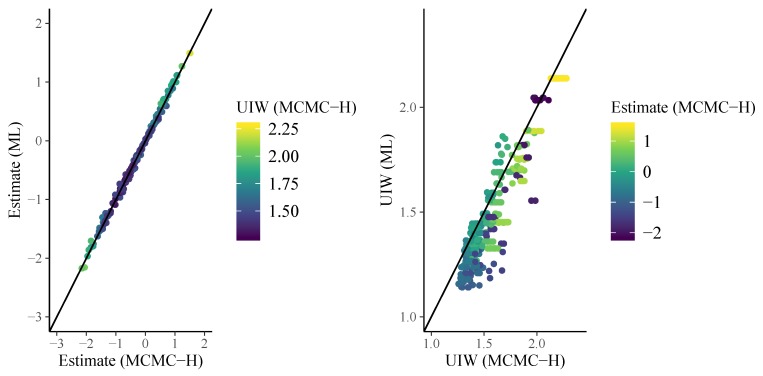
Comparison of 3PL person parameters: (Left) scatter plot of point estimates; and (Right) scatter plot of the associated 95% uncertainty interval widths (UIW).

**Figure 5:**
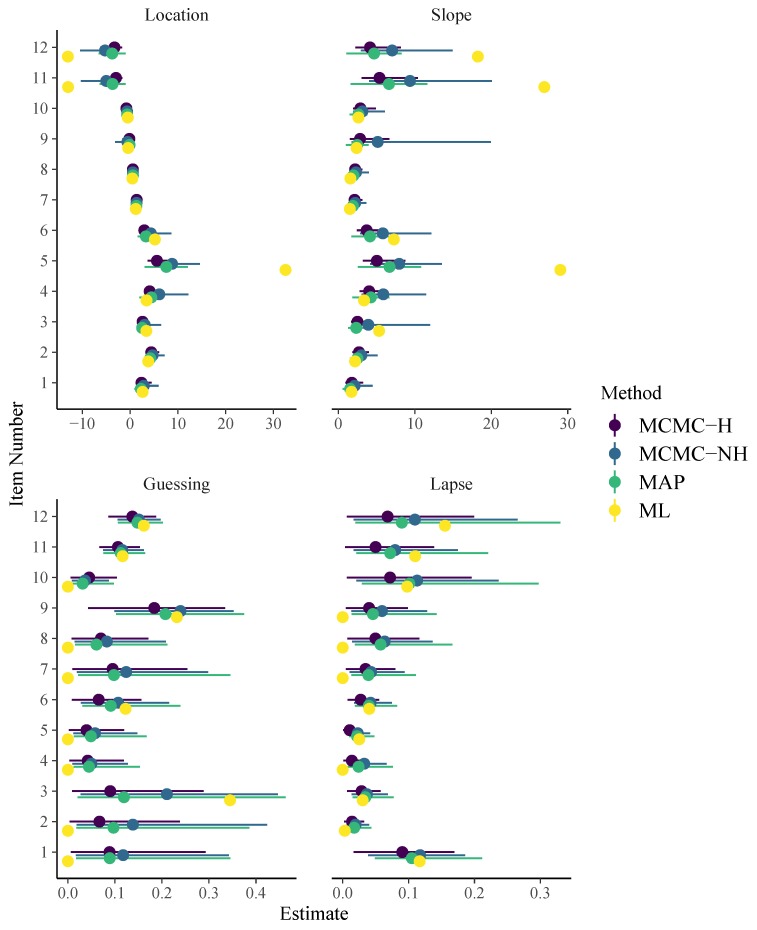
Item parameters of the 4PL model. Horizontal lines indicate 95% uncertainty intervals.

In Figure 4, I display person parameter estimates of the 3PL model. As we can see on the left-hand side of Figure 4, ML and MCMC-H point estimates align very closely. However, as displayed on the right-hand side of Figure 4, uncertainty estimates show some deviations, especially for more extreme point estimates (i.e., particularly good or bad performing participants). The brms formula for the 4PL model looks as follows: 
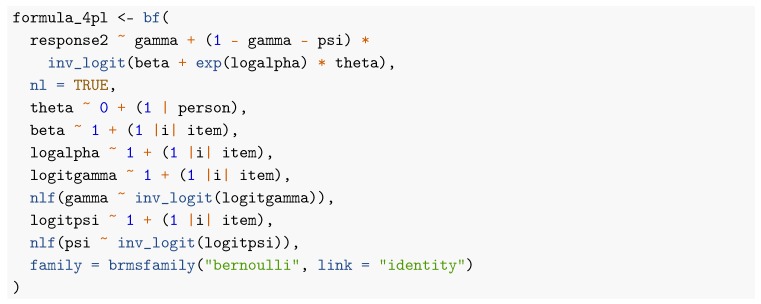


As displayed in Figure 5, item parameter estimates of the 4PL model differ strongly from each other for different methods. In particular, ML point estimates were more extreme and no uncertainty estimates could be obtained due to singularity of the information matrix. It is plausible that the 4PL model is too difficult to be estimated based on the given data via ML without further regularization. Moreover, the estimates obtained by MCMC-H and MCMC-NH differ noticeably for some item parameters in the way that MCMC-NH estimates tend to be more extreme and uncertain as compared to MCMC-H. This suggests that, for these specifically chosen hierarchical and non-hierarchical priors, the former imply stronger regularization.

### 3.2. Model Comparison

Next, I investigate the required model complexity to reasonably describe the SPM data. For this purpose, I apply Bayesian approximate leave-one-out cross-validation (LOO-CV; ref. Vehtari et al. 2019; Vehtari et al 2017; Vehtari et al. 2018) as a method for model comparison, which is closely related to information criteria (Vehtari et al 2017). I only focus on the MCMC-H models here. Results for the MCMC-NH models are similar (see Github for details). As shown in Table 1, 3PL and 4PL models fit substantially better than the 1PL and 2PL models, while there was little difference between the former two. Accordingly, in the interest of parsimony, I would tend to prefer the 3PL model if a single model needed to be chosen. This coincides with the conclusions of Myszkowski and Storme (Myszkowski and Storme 2018).

**Figure 6:**
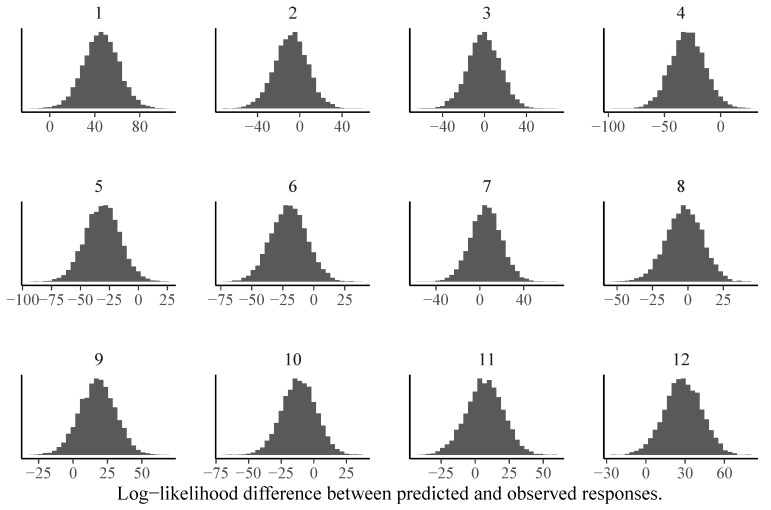
Item-specific posterior distributions of log-likelihood differences between predicted and observed responses for the 1PL model estimated via MCMC-H. If the majority of the posterior distribution is above zero, this indicates model misfit for the given item.

**Figure 7:**
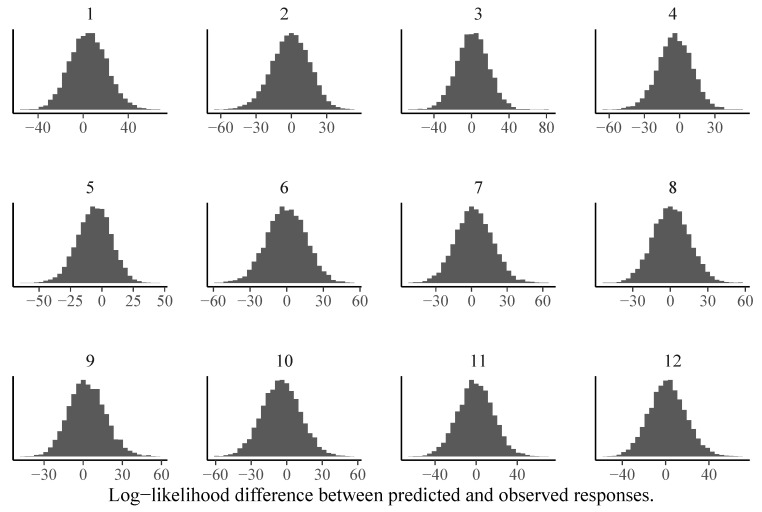
Item-specific posterior distributions of log-likelihood differences between predicted and observed responses for the 3PL model estimated via MCMC-H. If the majority of the posterior distribution is above zero, this indicates model misfit for the given item.

We can also investigate model fit using Bayesian versions of frequentist item or person fit statistics such as log-likelihood values (Glas and Meijer 2003). Independently of which statistic *T* is chosen, a Bayesian version of the statistic can be constructed as follows (Glas and Meijer 2003): First, the fit statistic is computed for the observed responses *y*. We denote it by T(y,p), where p=p(θ,ξ) is the model implied response probability defined in Equation (3). As *p* depends on the model parameters, the posterior distribution over the parameters implies a posterior distribution over *p*, which in turn implies a posterior distribution over T(y,p). Second, the fit statistic is computed for posterior predicted responses $y_{\mathrm{rep}}$ and we denote it by $T(y_{\mathrm{rep}},p)$. Since $y_{\mathrm{rep}}$ reflects the (posterior distribution of) responses that would be predicted if the model was true, $T(y_{\mathrm{rep}},p)$ provides a natural baseline for T(y,p). Third, by comparing the posterior distributions of T(y,p) and $T(y_{\mathrm{rep}},p)$, we can detect item- or person-specific model misfit. In Figure 6, we show item-specific log-likelihood differences between predicted and observed responses for the 1PL model. It is clearly visible that the assumptions of the 1PL model are violated for almost half of the items. In contrast, the corresponding results for the 3PL model look much more reasonable (see Figure 7).

**Figure 8:**
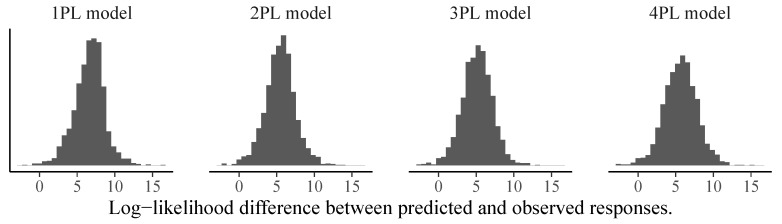
Person-specific posterior distributions of log-likelihood differences between predicted and observed responses for the 192nd person and different models estimated via MCMC-H. If the majority of the posterior distribution is above zero, this indicates model misfit for the given person.

**Figure 9:**
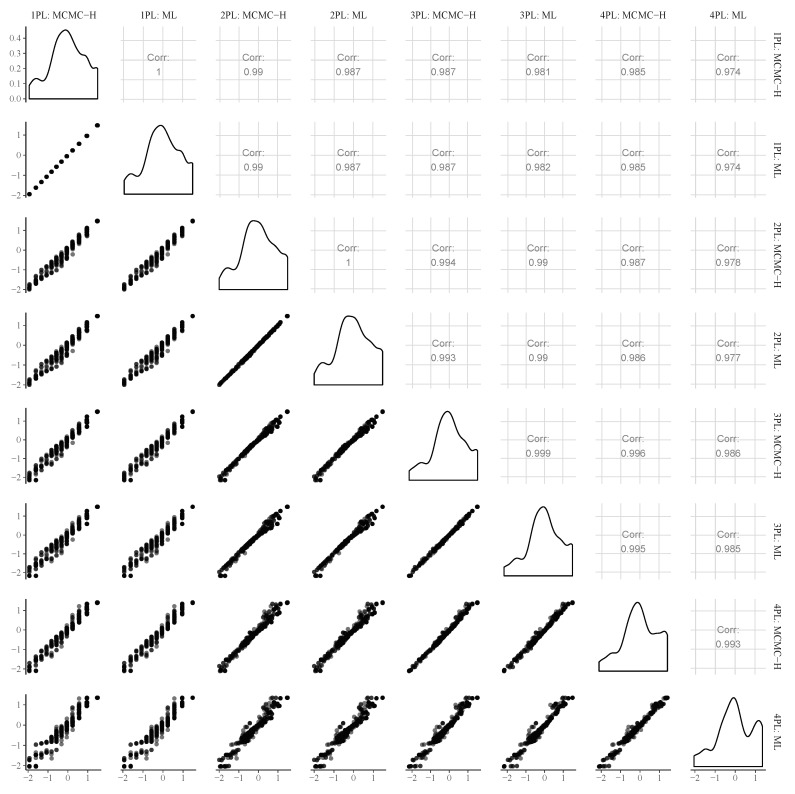
Scatter plots, bivariate correlations, and marginal densities of person parameters from MCMC-NH and ML models.

We can use the same logic to investigate person-specific model fit to find participants for whom the models do not make good predictions. In Figure 8, we show the predicted vs. observed log-likelihood differences of the 192nd person with response pattern (0,0,1,0,0,1,0,1,1,0,0,1). None of the models performs particularly well as this person did not answer some of the easiest items correctly (i.e., Items 2, 4, and 5) but was correct on some of the most difficult items (i.e., Items 8, 9, and 12). It is unclear what was driving such a response pattern. However, one could hypothesize that training effects over the course of the test played a role, which are not accounted for by all models presented here. To circumvent this in future test administrations, one could add more unevaluated practice items at the beginning of the test so that participants have the opportunity to become more familiar with the response format. Independently of the difference in model fit, person parameter estimates correlated quite strongly between different models and estimation approaches, with pairwise correlations exceeding r=0.97 in all cases (see Figure 9 for an illustration).

The time required for estimation of the Bayesian models with brms via MCMC ranged from a couple of minutes for the 1PL model to roughly half an hour for the 4PL model (exact timings vary according to several factors, for instance, the number of iterations and chains, applied computing machines, or the amount of parallelization). In contrast, the corresponding optimization methods (ML and MAP) required only a few seconds for estimation in mirt. This speed difference of multiple orders of magnitude is typical for comparisons between MCMC and optimization methods (e.g., Bürkner 2017). Clearly, if speed is an issue for the given application, full Bayesian estimation methods via MCMC should be applied carefully.

## 4. Discussion

In the present paper, I reanalyze data to validate a short version of Standard Progressive Matrices (SPM-LS; Myszkowski and Storme 2018) using Bayesian IRT models. By comparing out-of-sample predictive performance, I found evidence that the 3PL model with estimated guessing parameters outperformed simpler models and performed similarly well as the 4PL model, which additionally estimated lapse parameters. As specifying and fitting the 4PL model is substantially more involved than the 3PL model without apparent gains in out-of-sample predictive performance, I argue that the 3PL model should probably be the model of choice within the scope of all models considered here. That is, I come to a similar conclusion as Myszkowski and Storme (Myszkowski and Storme 2018) in their original analysis despite using different frameworks for model specification and estimation (Bayesian vs. frequentist) as well as predictive performance (approximate leave-one-out cross-validation (Vehtari et al 2017) vs. corrected AIC and $\chi^{2}$-based measures (Maydeu-Olivares 2013).

Even though I reach the same conclusions as Myszkowski and Storme (Myszkowski and Storme 2018) reached with conventional frequentist methods, I would still like to point out some advantages of applying Bayesian methods that we have seen in this application. With regard to item parameters, Bayesian and frequentist estimates showed several important differences for the most complex 3PL and 4PL IRT models. First, point estimates of items with particularly high difficulty or slope were more extreme in the frequentist maximum likelihood estimation. One central reason is the use weakly informative priors in the Bayesian models which effectively shrunk extremes a little towards the mean thus providing more conservative and robust estimates (Gelman et al. 2013). Specifically, for the 4PL model, the model structure was also too complex to allow for reasonable maximum likelihood estimation in the absence of any additional regularization to stabilize inference. The latter point also becomes apparent because no standard errors of the ML estimated items parameters in the 4PL model could be computed due to singularity of the information matrix. Even when formally computable, uncertainty estimates provided by the frequentist IRT models were not always meaningful. For instance, in the 3PL model, the confidence intervals of guessing parameters estimated to be close to zero were ranging the whole definition space between zero and one. This is clearly an artifact as maximum likelihood theory does not apply at the boundary of the parameter space and hence computation of standard errors is likely to fail. As such, these uncertainty estimates should not be trusted. Robust alternatives to computing approximate standard errors via maximum likelihood theory are bootstrapping or other general purpose data resampling methods (e.g., Freedman 1981; Junker and Sijtsma 2001; Mooney et al. 1993). These resampling methods come with additional computational costs as the model has to be repeatedly fitted to different datasets but can be used even in problematic cases where standard uncertainty estimators fail.

In contrast, due to the use of weakly informative priors, the Bayesian models provided sensible uncertainty estimates for all item parameters of every considered IRT model. MCMC and MAP estimates provided quite similar results for the item parameters in the context of the SPM-LS data and applied binary IRT models. However, there is no guarantee that this will be generally the case and thus it is usually safer to apply MCMC methods when computationally feasible. In addition, for the propagation of uncertainty to new quantities, for instance, posterior predictions, MCMC or other sampling-based methods are required. In the case study, I demonstrated this feature in the context of item and person fit statistics, which revealed important insides into the assumptions of the applied IRT models.

With regard to point estimates of person parameters, I found little differences between all considered Bayesian and frequentist IRT models. Pairwise correlations between point estimates of two different models were all exceeding r=0.97 and often even larger than r=0.99. This should not imply, however, that the model choice does not matter in the context of person parameter estimation (Loken and Rulison 2010). Although point estimates were highly similar, uncertainty estimates of person parameters varied substantially across model classes. Thus, it is still important to choose an appropriately complex model for the data (i.e., the 3PL model in our case) in order to get sensible uncertainty estimates. The latter are not only relevant for individual diagnostic purposes, which is undoubtedly a major application of intelligence tests, but also when using person parameters as predictors in other models while taking their estimation uncertainty into account. In addition, uncertainty estimates of Bayesian and frequentist models varied substantially even within the same model class, in particular for 3PL and 4PL models. Without a known ground truth, we have no direct evidence which of the uncertainty estimates are more accurate (with respect to some Bayesian and/or frequentist criteria), but I would argue in favor of the Bayesian results as they should have benefited from the application of weakly informative priors and overall more robust inference procedures for the considered classes of models. Overall, it is unsurprising that Bayesian methods have an easier time estimating uncertainty as it is more natural to do so in a Bayesian framework. We have also seen the important advantage of Bayesian methods that is their ability to more easily accommodate more complex models. However, we have also seen that, for simpler models, Bayesian and frequentist methods provide very similar results, which really speaks in favor of both methods and should highlight for the reader that both choices are valid options in this case and neither should be attacked. Developing this understanding seems necessary with the increased application of Bayesian methods and the accompanying arguments of whether this is a valid option.

The analysis presented here could be extended in various directions. First, one could fit polytomous IRT models that take into account potential differences between distractors and thus use more information than binary IRT models. Such polytomous IRT models were also fitted by Myszkowski and Storme (Myszkowski and Storme 2018) and demonstrated some information gain as compared to their binary counterparts. Fitting these polytomous IRT models in a Bayesian framework is possible as well, but currently not supported by brms in the here required form. Instead, one would have to use Stan directly, or another probabilistic programming language, whose introduction is out of scope of the present paper. Second, one could consider multiple person traits/latent variables to investigate the unidimensionality of the SPM-LS test. Currently, this cannot be done in brms in an elegant manner but will be possible in the future once formal measurement models have been implemented. For the time being, one has to fall back to full probabilistic programming languages such as Stan or more specialized IRT software that supports multidimensional Bayesian IRT models. According to Myszkowski and Storme (Myszkowski and Storme 2018), the SPM-LS test is sufficiently unidimensional to justify the application of unidimensional IRT models. Accordingly, the lack of multidimensional models does not constitute a major limitation for the present analysis.

In summary, I was able to replicate several key findings of Myszkowski and Storme (Myszkowski and Storme 2018). Additionally, I demonstrated that Bayesian IRT models have some important advantages over their frequentist counterparts when it comes to reliably fitting more complex response processes and providing sensible uncertainty estimates for all model parameters and other quantities of interest.

## Figures and Tables

**Table 1:** Bayesian Model comparison based on the leave-one-out cross-validation.

Model	ELPD	SE(ELPD)	ELPD-Difference	SE(ELPD-Difference)
4PL	−2544.7	42.6	0.0	0.0
3PL	−2547.8	42.8	−3.1	5.1
2PL	−2588.7	42.9	−44.0	9.5
1PL	−2655.0	43.8	−110.3	15.0

Note. ELPD, expected log posterior density; SE, standard error. Higher ELPD values indicate better model fit. ELPD differences are in comparison to the 4PL model.
